# Diagnostic ability of deep learning in detection of pancreatic tumour

**DOI:** 10.1038/s41598-023-36886-8

**Published:** 2023-06-15

**Authors:** M. G. Dinesh, Nebojsa Bacanin, S. S. Askar, Mohamed Abouhawwash

**Affiliations:** 1 Department of Computer Science and Engineering, EASA College of Engineering and Technology, Coimbatore, India; 2 Singidunum University, Belgrade, Serbia; 3 Department of Statistics and Operations Research, College of Science, King Saud University, P.O. Box 2455, 11451 Riyadh, Saudi Arabia; 4 Department of Computational Mathematics, Science and Engineering (CMSE), College of Engineering, Michigan State University, East Lansing, MI 48824 USA; 5 Department of Mathematics, Faculty of Science, Mansoura University, Mansoura, 35516 Egypt

**Keywords:** Cancer, Computational biology and bioinformatics

## Abstract

Pancreatic cancer is associated with higher mortality rates due to insufficient diagnosis techniques, often diagnosed at an advanced stage when effective treatment is no longer possible. Therefore, automated systems that can detect cancer early are crucial to improve diagnosis and treatment outcomes. In the medical field, several algorithms have been put into use. Valid and interpretable data are essential for effective diagnosis and therapy. There is much room for cutting-edge computer systems to develop. The main objective of this research is to predict pancreatic cancer early using deep learning and metaheuristic techniques. This research aims to create a deep learning and metaheuristic techniques-based system to predict pancreatic cancer early by analyzing medical imaging data, mainly CT scans, and identifying vital features and cancerous growths in the pancreas using Convolutional Neural Network (CNN) and YOLO model-based CNN (YCNN) models. Once diagnosed, the disease cannot be effectively treated, and its progression is unpredictable. That's why there's been a push in recent years to implement fully automated systems that can sense cancer at a prior stage and improve diagnosis and treatment. The paper aims to evaluate the effectiveness of the novel YCNN approach compared to other modern methods in predicting pancreatic cancer. To predict the vital features from the CT scan and the proportion of cancer feasts in the pancreas using the threshold parameters booked as markers. This paper employs a deep learning approach called a Convolutional Neural network (CNN) model to predict pancreatic cancer images. In addition, we use the YOLO model-based CNN (YCNN) to aid in the categorization process. Both biomarkers and CT image dataset is used for testing. The YCNN method was shown to perform well by a cent percent of accuracy compared to other modern techniques in a thorough review of comparative findings.

## Introduction

The most prevalent solid pancreatic cancer is pancreatic ductal adenocarcinoma (PDAC). Aggressive and challenging to cure, pancreatic cancer is the more general name for this disease. Pancreatic cancer has a meager endurance rate than other cancer types^1, 2^. Although there have been improvements in surgical methods, medication, and radiotherapy, the 5-year existence rate is still just 8.7%^3^. Pancreatic cancer is difficult to diagnose since most patients experience vague illnesses. While surgical resection plus chemotherapy gives the highest coincidental of life, with a 5-year survival rate of roughly 31.5%, only 10–20% of patients appear. Eighty to ninety percent of patients do not benefit from treatment because of widespread or regional metastases^4, 5^.

Compared to the incidence rates of other cancers with a higher death rate, such as lung, breast, and colorectal cancer, the overall incidence of malignancy is significantly lower. Therefore, age-based population screening is challenging because possible screening tests have low positive prediction performance, and there are many unnecessary assessments for individuals with false-positive findings. In addition, not many identified risk factors have a high penetrance for pancreatic cancer, making early identification of this illness difficult. For many years, the danger of pancreatic cancer has been evaluated based on family background, behavioral and physical lifestyle influences, and, more generally, systemic biomarkers and hereditary factors. This process began in the 1970s^6^. At this time, the serial pancreas-directed scan is performed on some associated with an increased risk due to family heritage or pathogenic genetic variations or cystic lesions of the pancreatic to detect slightly earlier pancreatic cancers.

However, accurate early diagnosis is still challenging and primarily reliant on imaging modalities^7^. Computed tomography (CT) is the most frequent imaging sense modality for the first examination of alleged pancreatic cancer^8, 9^, outranking ultrasonography, MRI, and endoscopic ultrasonography. Subclinical people with a high risk of developing pancreatic cancer can also be screened with CT scans. The average survival time for patients with pancreatic cancer identified accidentally during an imaging scan for a particular condition is significantly longer than those who presented with clinical symptoms^10^. CT has a 70–90 percent sensitivity for detecting pancreatic adenocarcinoma^11^. Thin-section contrast-enhanced dual-phase multidetector computed tomography is the modality of choice for diagnosing pancreatic cancer^12^.

The main objective of this research is to predict pancreatic cancer early using deep learning and metaheuristic techniques. Also, Analyze both biomarkers and CT image datasets to test the performance of the developed models. Specifically, the research aims to develop a deep learning and metaheuristic techniques-based system to predict pancreatic cancer early by analyzing medical imaging data, particularly CT scans. The objective is to identify vital features and cancerous growths in the pancreas using Convolutional Neural Network (CNN) and YOLO model-based CNN (YCNN) models.

Recent advances in deep neural networks and rising healthcare demands have shifted the focus of AI research toward CAD systems. Some early breakthroughs have been seen in using deep learning to evaluate radiological images. A deep learning-aided decision-making approach has been utilized to help diagnose lung nodules and skin tumors^13, 14^. Given the severity of pancreatic cancer, it is essential to work on creating CAD systems that can tell cancerous from noncancerous tissue. Consequently, creating a sophisticated discriminating mechanism for pancreatic cancer is essential. A convolutional neural network (CNN) can extract characteristics from the image by probing the local spatial correlations in a picture. CNN models have been able to effectively address a wide variety of challenges relating to the classification of pictures^15^. In this paper, we used clinical CT scans to show that a deep learning approach can accurately classify pancreatic ductal adenocarcinoma, as confirmed by a pathologist.

The following is the structure of this paper: In Section “Related works”, we discuss the work done on supervised and unsupervised learning to diagnose pancreatic cancer. In Section “Proposed YOLOv3 based CNN methodology for pancreatic cancer classification”, we will discuss the algorithm that we use for deep understanding. In the previous section (Section “Dataset”), we discussed the experimental set and the datasets utilized for the investigation. The discussion of the results of the experiment can be found in Section “Sample code and Implications”. Discussions and closing comments are included in the last section, numbered 6.

## Related works

This section offers a comprehensive analysis of the many categorization schemes for pancreatic tumors that have previously been published. A CNN classifier was developed by Ma et al.^16^ to detect pancreatic cancers in CT data automatically. A dataset of 3494 CT scans was obtained from 3751 CT scans of 190 people with typical pancreatic cancer and 222 with pathologically proven pancreas tumors. This dataset was used to develop a CNN algorithm. They extracted three datasets from the picture, calculated the approach concerning ternary classifiers, and evaluated the algorithm's efficacy in specificity, accuracy, and sensitivity with tenfold cross-validation.

In^17^, an eightfold cross-validation approach is used to measure performance after a CNN-based DL technique was applied to CECT images to get three methods (arterial or venous, arterial, and venous). When evaluating the TML and DL algorithms for predicting the pathological grading of pNEN, the optimal CECT picture is used for comparison. Quantitative and qualitative CT data were also used to assess radiologists' efficiency. Using an eightfold cross-validation procedure, we could estimate the best DL approach for scanning a separate testing set of 19 individuals from Hospital II using different scanners. For the challenging task of pancreatic segmentation, Fu et al.^18^ introduced a novel pancreatic segmentation network that initially extends the RCF described to the edge detection domains. The divulged connectivity carried out per-pixel categorization by meticulously considering objects' multi-resolution extensive contexture data (pancreas). This was possible using a multilayer up-sampling design instead of each level's most fundamental up-sampling activities. This network was also trained and fed with CT images, producing a productive outcome.

Manabe et al.^19^ calculated a modified CNN technique to boost the efficiency of medical pictures. They changed the AlexNet technique based on convolutional neural networks to work with a 512-by-512 input space. Maximum pooling and convolutional layers both had their filter sizes decreased. Many other approaches were tested and developed using this modified CNN. Improved Convolutional Neural Network (CNN) estimates for pancreatic absence/presence CT image classification were made. Total accuracy measured on test photos not used to train the Resnet was also correlated.

Malignancy classification of lung nodules benefited from the knowledge of many high-level picture characteristics. Eighty-two percent of lobulated nodules, ninety-three percent of ragged nodules, ninety-seven percent of heavily spiculated nodules, and one hundred percent of halo nodules were malignant in a dataset studied by^20^. Automatic identification of characteristics and kinds of lung nodules was investigated in^21^. This project aimed to categorize six distinct forms of nodules (solid, non-solid, part-solid, calcified, perivisceral, and spiculated nodules). This method relies on 2D CNN, which is inadequate for assessing lung nodule malignancy. Further, benign status was assigned to 66% of the round nodules.

By using massive volumes of imaging data, artificial intelligence has the potential to aid radiologists in the early identification of PDAC. In particular, CNNs belong to the family of AI algorithms known as deep learning models, and they have demonstrated excellent accuracy in the image-based diagnosis of several cancers^22, 23^. With the scan as input, CNNs routinely extract features useful for the diagnostic job through a chain of convolutions and pools. Attempts to automate the diagnosis of PDAC have recently shifted focus to deep learning models^24–29^. However, the majority of studies conduct binary classification, determining whether or not a given input picture contains cancer, and do not also localize lesions at the same time. Not only that, but just one research^27^ reported the model’s performance for tumors less than 2 cm in size. In contrast, most papers paid little attention to these early-stage lesions.

Transfer learning is a machine learning technique where knowledge gained from solving one problem is applied to predict pancreatic cancers^30, 31^. In this case, pre-trained deep learning models trained on large image datasets are fine-tuned to detect cancerous lesions in pancreatic images. The aim is to improve the detection of pancreatic cancer at an early stage, which is critical for better patient outcomes. Also, authors in^32^ have suggested that deep learning has more effective in predicting pancreatic cancer when compared to other ML techniques. The article^33^ highlights the poor prognosis of pancreatic cancer due to late diagnosis and the importance of early detection and identification of molecular targets for treatment. It also suggests that screening and surveillance in high-risk groups, combined with new biomarkers, may offer new strategies for risk assessment, detection, and prevention of pancreatic cancer.

The paper^34^ describes the development of a machine-learning algorithm based on changes in 5-hydroxymethylcytosine signals in cell-free DNA from plasma for early detection of pancreatic cancer in high-risk individuals. The ML algorithm shows high specificity and sensitivity in distinguishing pancreatic cancer from noncancer subjects, with a sensitivity of 68.3% for early-stage cancer and an overall specificity of 96.9%. The article^35^ describes a study investigating the link between serum proteins and early-stage cancer detection. The study found that a positive history of alcohol consumption can diminish the sensitivity of serum protein-mediated liquid biopsy in detecting early-stage malignancies, resulting in a 44% decline in sensitivity. A grouped neural network is proposed in^36^ for early diagnosis of pancreatic cancer using laboratory health tracking. The work^37^ describes a study that aims to develop new methods to simplify the large volume of patient medical records to improve clinical decision-making. The study uses deep-learning architectures to create simplified patient state representations that are predictive and interpretable to physicians.

The radiology department has most cancer diagnosis tests using machine and deep learning algorithms^38, 39^. The prediction accuracy is relatively achieved higher using these machine learning algorithms. Various medical diagnosis of pancreatic symptoms is tested in the radiology department^40^. The article^41^ induces multiple applications on the radiology side that have used AI. This paper discusses various applications for pancreatic cancer prediction.

The study proposes that this beneficial effect of long noncoding RNA p21 on endothelial repair is mediated by a pathway involving three protein molecules: SESN2, AMPK, and TSC2^42^. The study proposes that the inhibitory effect of homocysteine on pro-insulin receptor cleavage is caused by a process called cysteine-homo cysteinylation. Cysteine is another amino acid containing a sulfur atom, and homocysteine can form disulfide bonds with cysteine residues on proteins, altering their function^43^. It involves using hyperpolarization techniques to increase the sensitivity of NMR, which allows for the detection of rare and subtle interactions between molecules^44^. Drug delivery systems are methods for delivering drugs to specific targets in the body, such as diseased cells or tissues, while minimizing the potential for side effects^45^. The method also incorporates DS evidence theory, a mathematical framework for combining different types of evidence and uncertainty in decision-making^46^. Deep learning is a type of machine learning that uses artificial neural networks to learn from large amounts of data and make predictions or classifications^47^. The study also identified some critical factors that influence the thermal behavior of solid propellants, such as the presence of additives and the effects of moisture^48, 49^. The study results showed that silencing GTF2B expression led to a significant decrease in the proliferation of A549 cells, suggesting that GTF2B plays a role in promoting cell growth^50, 51^.

The study evaluated the proposed method's effectiveness using a large lung CT image dataset. It showed that it outperformed existing image retrieval methods regarding accuracy and computational efficiency^52, 53^. The study evaluated the effectiveness of the proposed method using a large dataset of CT image sequences. It showed that it significantly improved retrieval time and accuracy compared to existing methods for mobile telemedicine networks^54^. ViT-Patch is a deep learning model based on a type of neural network called a transformer that has recently shown state-of-the-art performance on a range of image classification tasks^55^. The method involves first transforming the input images into a sparse representation using a sparse dictionary^56^.

The researchers evaluated the efficacy of their surface-functionalized biomaterials using in vitro and in vivo experiments^57^. The researchers also demonstrated the feasibility of using phased array technology to generate and detect guided waves in curved plates, which could have critical applications in structural health monitoring and damage detection^58, 59^. The results showed a significant association between the health status of family members and the health behaviors of other family members. Specifically, having a family member with good health was associated with a higher likelihood of engaging in healthy behaviors such as regular exercise and not smoking^60, 61^. OCT is a non-invasive imaging technique that uses light waves to capture detailed images of the retina, and it is commonly used for diagnosing and managing ERM^62, 63^. The results showed that treatment with the therapeutic aptamer significantly increased bone formation in the mice with OI without increasing their cardiovascular risk^64, 65^.

This study utilized spectral domain optical coherence tomography (SD-OCT) to examine the postoperative outcomes of vitrectomy in highly myopic macular holes^66^. Sclerostin is a naturally occurring protein that inhibits the activity of cells responsible for bone formation called osteoblasts. It is crucial in regulating bone metabolism and preventing excessive bone growth^67^. It targets specific proteins, known as immune checkpoints, that inhibit immune cell activity^68^. Nanotherapeutic platforms refer to nanoscale materials that can be utilized for therapeutic purposes. In this case, the focus is on metal-based nanoparticles and their potential applications in treating bacterial infections^69^. The researchers used SRS microscopy to acquire high-resolution prostate core needle biopsies images. These images captured the distribution and composition of different biomolecules within the tissue, enabling a detailed analysis of the cancerous features^70^.

From the literature study, it is shown that deep learning algorithms are well suited to the diagnosis of pancreatic cancers. This research contributes by using deep learning and metaheuristic model to predict pancreatic cancer earlier.

1. First, CNN is used to classify the pancreatic cancer images
2. Data pre-processing and segmentation process is done using sail fish optimizer.
3. Finally, a novel YOLO-based CNN model is used to predict cancer objects and classify cancer patients with high accuracy

The significant limitations of literature studies as follows

1. Studies mentioned in the discussion may have limitations regarding evaluating their proposed techniques. For example, the review might be limited to a specific dataset or may not involve a large and diverse sample size, which could affect the generalizability of the results.
2. Many studies primarily focus on the binary classification of pancreatic cancer (presence or absence) and may not adequately address the detection and characterization of early-stage lesions. Early detection improves prognosis, but only some papers address this.
3. IWhile deep learning models have been employed for cancer detection, most studies do not simultaneously focus on localizing lesions within the pancreas. Localization is essential for accurate diagnosis and treatment planning; more research is needed in this area.
4. Only one study reports the model’s performance for tumors smaller than 2 cm. The detection and characterization of small tumors are critical for early diagnosis, and further investigation is needed to assess the effectiveness of the proposed methods for these lesions.
5. The studies mentioned utilize various image datasets for training and evaluation. It is essential to consider the potential biases and limitations in these datasets, such as variations in image quality, patient demographics, and imaging protocols, which may affect the generalizability of the results.
6. Some studies may need more clinical validation or evaluation in real-world settings. Further research is necessary to assess the effectiveness of the proposed techniques in clinical practice and their integration into existing diagnostic workflows.

## Proposed YOLOv3 based CNN methodology for pancreatic cancer classification

**Figure 1:**
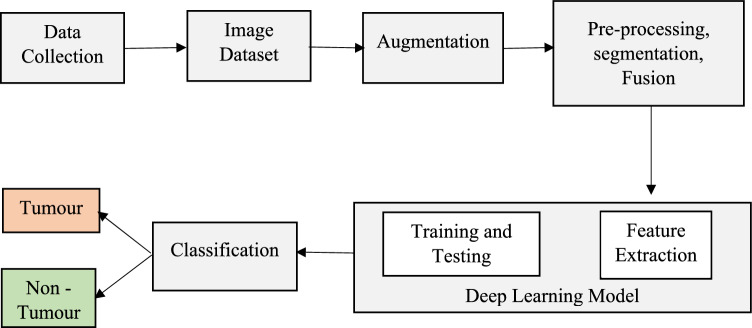
Overall framework of the pancreatic cancer model.

The process of the model is illustrated in Fig. 1. During training, we determined which of the initial CT scans of the abdomen had accurate pictures for diagnosis. Following the augmentation of the data, we created a deep-learning model consisting of three linked sub-networks. These models are commonly utilized in medical picture recognition due to their established effectiveness^29, 71^. Images of the pancreas that include it may be recognized with the help of ResNet50. The transverse plane CT scans shown in Fig. 1 do not have the pancreas and are not directly utilized in the YCNN model diagnosis. It does it by making predictions on each pixel of the picture, which produces binary values for the pancreatic segmentation.

This research incorporated texture characteristics of the pancreas into the segmentation outcome so that the future sub-network would have a more robust diagnostic base. This was done during the subsequent image fusion process. ResNet50, the last neural network in the YCNN model, is employed to determine whether or not a patient has a pancreatic tumor. The quantity of discrepancy between the production of the neural network and the label is used to calculate the loss function, and the back-propagation approach is applied to calculate how each gradient weight must be upgraded. The loss function is based on the level of divergence that persists between the production of the human brain and the classification. After analyzing the data, we chose the weights that would result in the least amount of data being lost and then locked them down for later application to the testing dataset.

In Fig. 1, we depict our unique and practical framework for tumor identification. The network's core is an amalgamation of Feature Pyramid Networks (FPN) and P-CNN, and its contributions are comprised of three parts: augmented FPNs, SAFF, and a Dependencies Computation Module. First, we use a convolutional neural network (CNN) to extract features from the pre-processed CT images. Next, we construct the feature pyramid using up-sampling and horizontal connections. Second, a bottom-up approach is set up to make the transmission of low-level localization information more efficient, which improves the overall feature hierarchy and, in turn, the detection performance. Finally, we use a Region Proposal Network (RPN) at each tier to create proposals before employing Feature Fusion to increase the associated region of interest and encode richer background information across various balances. To further capture each proposal's interdependencies with its surrounding tissues, we run the Dependencies Computation Module.

### Segmentation

In order to segment images, we use a method based on Kapur's thresholding (SFO-KT)^72^ and the sailfish optimizer. During the image decomposition method, the pre-processed photograph is used as input by the SFO-KT technique in order to find the problematic regions in the CT scan. Therefore far, it has seen the most useful in determining the best threshold for histogram-based picture segmentation. At first, the entropy criteria were proposed for bilevel thresholding, much like the Otsu model. It is expressed in^72^,

1 $${E}_{0}= -\sum_{i=0}^{t}\left(\frac{{YR}_{i}}{{\omega }_{0}}\right) \mathrm{ln}\left(\frac{{YR}_{i}}{{\omega }_{0}}\right), {E}_{1}= -\sum_{i=t+1}^{L-1}\left(\frac{{YR}_{i}}{{\omega }_{1}}\right) \mathrm{ln}\left(\frac{{YR}_{i}}{{\omega }_{1}}\right)$$

2 $${f}_{kapur}\left(t\right)={E}_{0}+{E}_{1}$$

The equation represents the calculation of the Kapur's entropy function, denoted as f kapur(t), based on two probabilities distributions of a discrete variable YR. The variable YR represents a set of non-negative real numbers, and it can be interpreted as the ratio of two positive quantities ω_0_ and ω_1_.

The formula is split into two terms, E0 and E1, which are the entropy of YR for two different intervals. The first term, E0, is the entropy of YR for the interval [0, t], and it is calculated by summing over all YR values in that interval. Specifically, for each YR value in that interval, we calculate its probability as YR_i_ / ω_0_, where ω_0_ is a reference quantity, and then we take the logarithm of this probability and multiply it by the probability itself. This process is done for all YR values in the interval, and the results are summed to obtain E_0_.

The second term, E_1_, is the entropy of YR for the interval [t + 1, L-1], where L is the total number of YR values. It is calculated in a similar way as E_0_, except that we use ω_1_ as the reference quantity instead of ω_0_.

Finally, the Kapur's entropy function, f_kapur(t), is obtained by adding E_0_ and E_1_ together. The parameter t is a threshold value that splits the YR values into two groups, [0, t] and [t + 1, L-1], and the function f kapur(t) measures the total entropy of YR for these two groups. The optimal value of t is the one that minimizes f kapur(t), and it is used as a criterion for selecting the best threshold value for classification or segmentation tasks.

Next, threshold values of kapur entropy is optimized using sail fish optimizer (SFO). SFO is metaheuristic approach which is based on sail fish attack alteration strategy. The position of the ith sailfish in the kth search round was denoted by SA_i,k_ and its corresponding fitness was evaluated as f(SA_i,k_). In the SFO technique, sardines also played a significant role. They were represented as a school moving through the search space, and the position of the i_th_ sardine was denoted by SR_i_, with its fitness evaluated as f(SR_i_).

The elite sailfish, possessing the optimal position, was selected during the SFO technique to influence the manoeuvrability and acceleration of sardines under attack. Additionally, the optimal position of any injured sardines from previous rounds was chosen for collaborative hunting by the sailfish to avoid selecting previously discarded solutions. These elite sailfish and injured sardines were designated as Y_new_ SA_i_, which represents^72^ an upgraded solution dependent on subsequent iterations, is represented as,

3 $${Y}_{{new}_{SA}}^{i}={Y}_{{elite}_{SA}}^{i}-{X}_{i }*(random\left(\mathrm{0,1}\right)*\left(\frac{{Y}_{{elite}_{SA}}^{i}-{Y}_{{injured}_{SR}}^{i}}{2}\right)-{Y}_{{current}_{SA}}^{i}$$

4 $$\mathrm{where}, {X}_{i }=2*rand\left(\mathrm{0,1}\right)*SRD-SRD$$

where SRD denote sardine density.

5 $$SRD=1-\left(\frac{{A}_{SA}}{{A}_{SA}+{A}_{S}}\right)$$

In this, A represent amount sailfish and sardine. Then the new position of sardine is updated. Next, attack power of sailfish is computed with new position. Sardine upgrade to new position. if sardine is hunted, then fitness is superior to sail fish. Once the value is optimized then the segmentation is performed.

### Feature pyramid network

**Figure 2:**
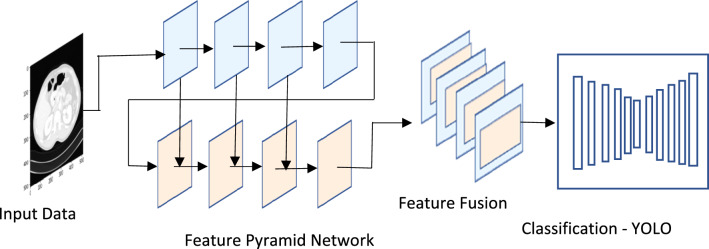
The proposed CNN architecture.

CNNs can glean semantic information throughout the feature extraction process. Similarly, high-level feature maps have a very positive response to global characteristics, making them ideal for spotting massive objects. However, because the tumor is so tiny in CT images, the successive pooling layers risk distorting the feature maps' spatial information. In addition, tumour recognition relies heavily on low-level exact localization data; however, the propagation effect is influenced by the lengthy (over a hundred layers) data communication channel in FPN. For this purpose, we construct an Augmented Feature Pyramid working from the bottom up. Initially, we create $Q1,Q2,Q3, and Q4$ using FPN. Then, starting at $Q1$, the enhanced route is formed, and $Q1$ is immediately utilised as $R1$ without further transformation. The next step is to execute a $3\times 3$ convolutional operator with stride 2 on a higher resolution feature map ${R}_{i}$ in order to shrink the size of the map. After that, we combine the down sampled feature map with another, coarser feature map, ${Q}_{i+1}$, by summing their respective elements. We then apply a second $3\times 3$ convolutional operator to each fused feature map to produce ${R}_{i+1}$ for subsequent feature map creation. This procedure is repeated until the level $Q4$ is reached. A fresh $R1,R2, R3, and R4$ Augmented Feature Pyramid is thus obtained. The Fig. 2. displays the proposed YCNN construction.

### Feature fusion

As a result of focusing on a single level for all of the actions after obtaining the suggested areas with RPN, certain potentially relevant details from lower levels are lost in the process. To fully use context information at many scales, we propose a Self-adaptive Feature Fusion module that integrates hierarchical feature maps from several layers. Each proposal's level ${R}_{m}$ of the Augmented Feature Pyramid is formally determined by assigning the ROI with dimensions $w$ and $h$.

6 $$m = min\left( {R_{max ,} max\left( {\left\lfloor {m_{0} + log_{2} \left( {\sqrt{\frac{wh}{I}} } \right)} \right\rfloor ,R_{min} } \right)} \right)$$

$\mathrm{I}$ denotes the input image size 224. Through an examination of CT scans, clinicians are able to locate tumours by studying the image's universal context, local geometric edifices, shape changes, and most importantly, spatial interactions with adjacent tissues. To calculate the retort at a given point, which is a biased sum of the characteristics at all positions on the expanded region $Q$, we make use of the Dependencies Computation Module. One of the most helpful pieces of data for detecting tumours may be accessed by performing this process, which allows the network to focus more on connections and dependencies at various scales, from the local to the global. To be more precise, the whole Addictions Computation Module is well-defined as follows, with $i$ as the input.

7 $${Y}_{l}=softmax(\varnothing ({Y}_{l},{Y}_{m}))H({Y}_{m})$$

8 $$softmax(\varnothing ({Y}_{l},{Y}_{m}))=\frac{e(\varnothing ({Y}_{l},{Y}_{m}))}{\sum_{m=1}^{M}e(\varnothing ({Y}_{l},{Y}_{m})}$$

**Figure 3:**
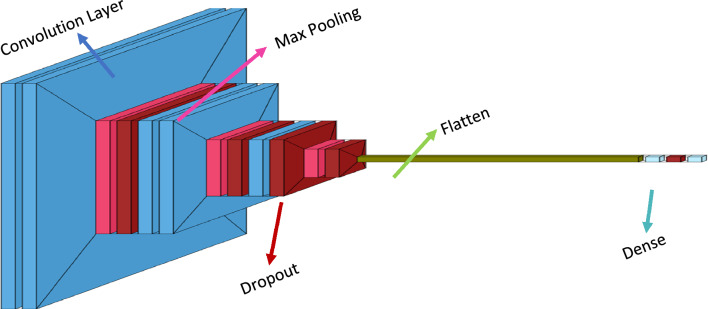
Construction of CNN model.

We created a CNN model to classify CT scan images for use in the early identification of pancreatic cancer. As shown in Fig. 3, our suggested CNN model has the following architectural layout. With three convolutional layers and a fully linked layer, our model was somewhat complex. Evey convolutional layer was followed by a weaker than expected max-pooling layer, a rectified linear unit (ReLU) layer that applied an activation function, and a batch normalisation (BN) layer to constrain the layer's output results. To further minimize the dimensionality of the feature values sent into the fully connected layer, we also implemented an average-pooling layer beforehand. To avoid overfitting and overspecialization, we chose a 0.5 percentage point dropout rate between both the median and fully linked layers. In addition, we attempted implementing a Spatial Dropout between each max-pooling layer and the convolutional layer that followed, however this led to a decrease in overall performance. This is why Spatial Dropout was not used. The network accepts the CT image's pixel values as input and returns the likelihood that the picture belongs to a certain class as output. Our model was gradually given the CT scans. Every layer receives as input the numbers generated by the layer above it. Layers process the input values by applying various transformations before sending them on to the following layer.

Our model was trained on the training set with a mini-batch size of 32 using a dataset with n target classes. The loss between our model's predictions and the true outcomes was computed using the cross-entropy loss function at the end of each training cycle. This reduction influenced Adam's optimization of weight modifications to our CNN model. After making changes to the model, we evaluated it based on its performance on the validation data. Our model was trained for up to 100 iterations before the one with the greatest accuracy on the validation set was chosen. Our methods were tested using a cross-validation procedure with a tenfold increase in sample size. Each set of photos from each stage was randomly split into 10 groups , of which 8 were used for training, 1 for validation, and 2 for testing the model. This was done ten times, with each "fold" serving as the test set once. Results were noted as being around average. We measured the accuracy, precision, and recall of our CNN model on the test sets.

### Classification model

In order to advance the accuracy of our finding, we made advantage of the overall design of YOLOv3, with DarkNet53 serving as the network's backbone and a three-layer spatial pyramid serving as the neck. The BCE Loss function was utilised as the target loss function in the detection head, and a branch and loss function that was particularly optimised was added to the original YOLOv3 implementation. YOLOv3 model is used in detection of cancer as an object and classify the image. Because the accuracy of the classification was more significant than the detection area for the early cancer detection, we decided to provide a bigger weight to the loss of classification. We made to start generating the network configuration in a random fashion. This was done to ensure that the activation function insights for each layer at the starting of the training stage were within a reasonable interval, which was necessary to ensure that the network would converge quickly.

**Figure 4:**
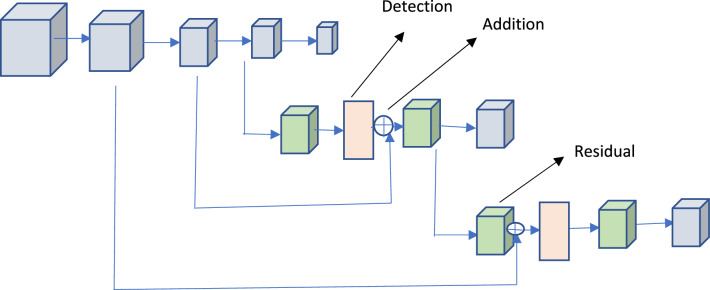
The sample architecture of YOLO Model used in our proposed model.

Since the dataset we utilized was much smaller than the data used in the YOLOv3 network, it was possible that overfitting would occur if we had learned effectively with the provided boundaries. Therefore, in order to determine the characteristics of the DarkNet53 backbone network, Upon first, we did some preliminary training on the Image Net's image recognition job and the dataset's object classification task. After that, we added a three-layer pyramid detecting neck and fine-tuned it using the data set for early cancer. As can be seen in Fig. 4, we normalized the photographs from Image Net and the data sources by using the range and mean of the slightly earlier tumor training set to best align the learning rate with the early tumor data set. This lets us go as close as possible to fitting the model parameters to the early cancer data set. We utilized 64 images for each iteration of the network's fine-tuning process, with entries measuring 224 by 224 pixels. As a result of the limited memory available on the GPU, a batch was split into 32 divisions. The total number of epochs that were performed was one hundred, with the first two epochs serving as the warm-up training and employing a cosine learning rate of 0.01 for each epoch. After the warm-up, the learning rate dropped to 0.001 each epoch. We worked to provide context to the data, increasing their value.

## Dataset

### Image dataset

**Figure 5:**
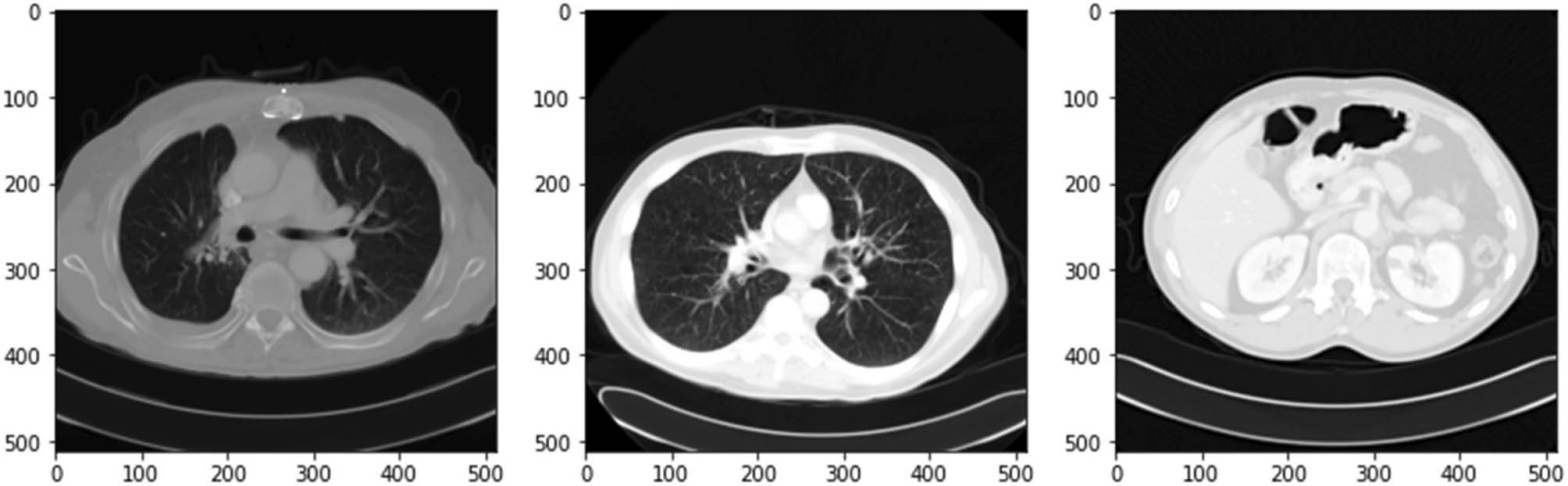
Sample CT scan images from the dataset.

In the period between June 2017 and June 2018, a dataset of 3494 CT pictures was gathered from 222 patients with pathologically proven pancreatic cancer, while a dataset of 3751 CT images was gathered from 190 individuals with healthy pancreas, and using these images, a CNN model was developed. The sample images are shown in the Fig. 5.

We divided these pictures into three phases-based datasets, tested the method using tenfold cross validation for binary classification (i.e., cancer or not) as shown in the Table 1.

**Table 1:** Image dataset information.

Particulars	Data
Total pancreatic cancer images	3494
Patient with pancreatic cancer	222
Total healthy pancreas images	3451
Patient with healthy pancreas	190
Thickness	5.0 mm

### Urinary biomarkers

Urinary biomarkers have been investigated for their potential role in the detection of pancreatic cancer. Pancreatic cancer is often diagnosed at an advanced stage when treatment options are limited, and the prognosis is poor. Therefore, identifying reliable and non-invasive biomarkers for early detection is critical for improving outcomes.

Several urinary biomarkers have been studied for their potential in detecting pancreatic cancer, including:

1. CA 19–9: A glycoprotein that is often elevated in pancreatic cancer patients and is currently used as a biomarker in clinical practice.
2. MUC1: A transmembrane mucin protein that has been found to be overexpressed in pancreatic cancer.
3. Osteopontin: A glycoprotein that has been shown to be overexpressed in pancreatic cancer and can be detected in urine.
4. Tumor-associated trypsin inhibitor (TATI): A protein that is often elevated in pancreatic cancer patients and has been investigated as a potential biomarker.
5. Human epididymis protein 4 (HE4): A glycoprotein that has been found to be overexpressed in pancreatic cancer and can be detected in urine.

This research uses LYVE1 (Lymphatic Vessel Endothelial Hyaluronan Receptor 1), REG1B (Regenerating islet-derived protein 1 beta), TFF1 (Trefoil factor 1) and REG1A (Regenerating islet-derived protein 1-alpha) biomarker as a dataset.

**Figure 6:**
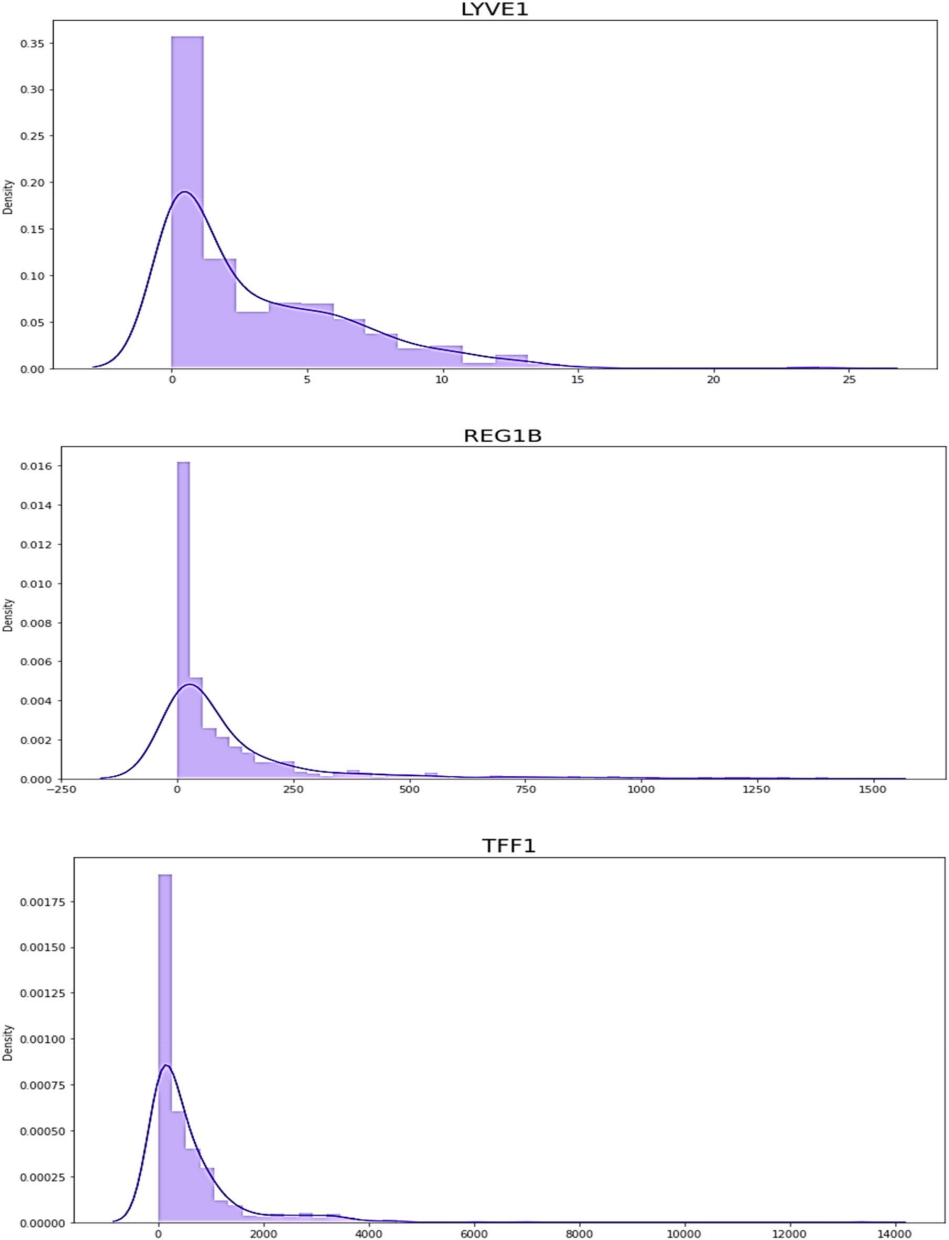
the circulation of data in biomarkers dataset.

LYVE1 has been investigated as a potential target for cancer therapy, as its expression has been found to be upregulated in various types of tumors, including breast, lung, and pancreatic cancer. A study published in the journal Pancreas in 2014 found that REG1B was significantly elevated in the serum of pancreatic cancer patients compared to healthy controls and patients with pancreatitis. Another study published in the same journal in 2017 found that REG1B levels were higher in the urine of pancreatic cancer patients compared to healthy controls and patients with chronic pancreatitis. A study published in the journal PLOS ONE in 2017 found that TFF1 levels were significantly higher in the serum of pancreatic cancer patients compared to healthy controls and patients with pancreatitis. Another study published in the journal Oncotarget in 2018 found that TFF1 levels were higher in the urine of pancreatic cancer patients compared to healthy controls and patients with chronic pancreatitis. REG1A has been shown to be a prognostic indicator of pancreatic cancer, with higher levels of REG1A associated with poorer outcomes. The Fig. 6 shows data of biomarkers present in the used dataset.

**Figure 7:**
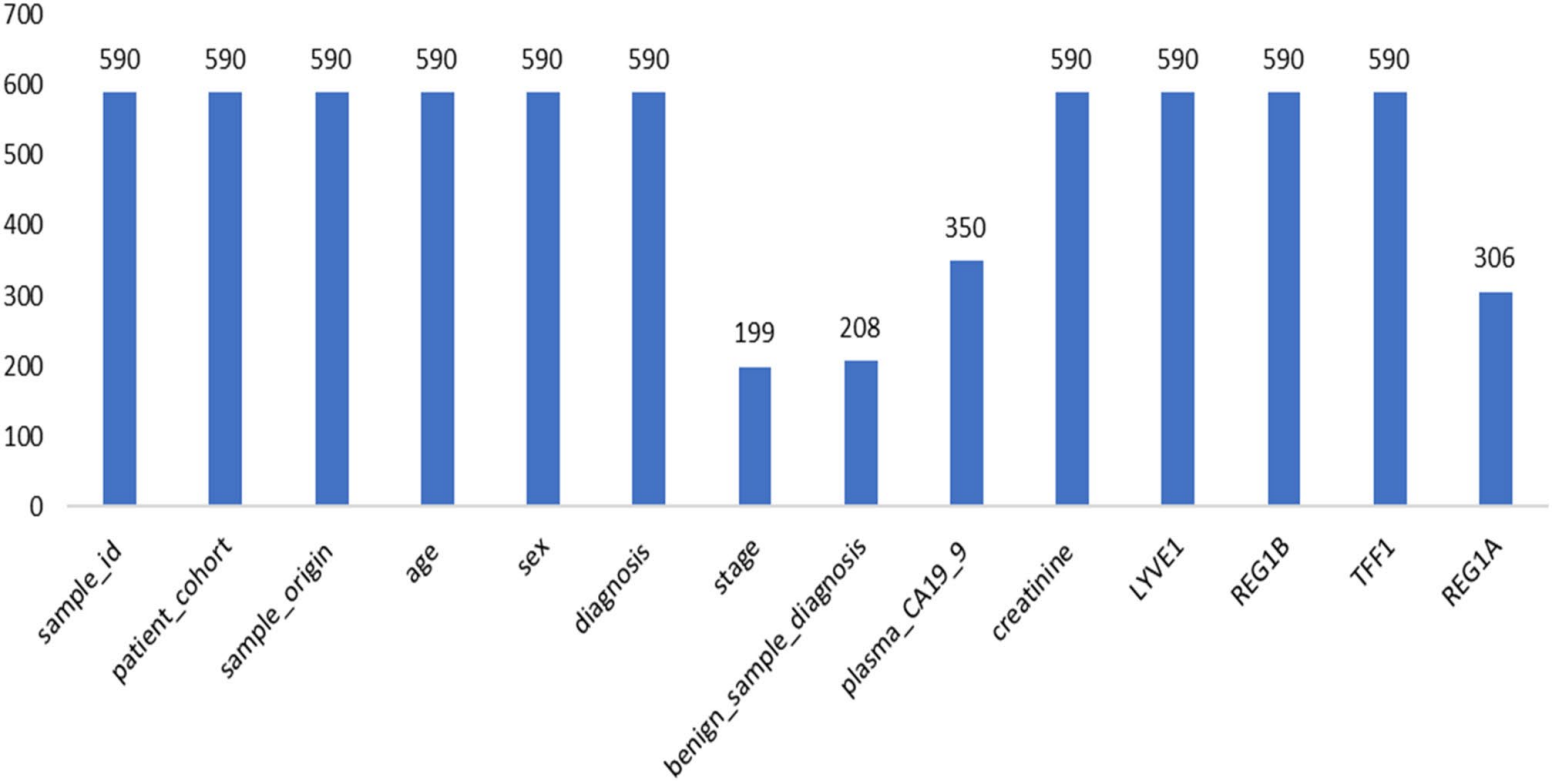
The dataset information.

They collected biomarkers from the urine of three distinct patient populations:

- Health indicators
- Pancreatic ductal adenocarcinoma patients, malignant pancreatic environments
- They were coordinated by age and sex when accurate. The purpose was to discover a reliable method of diagnosing pancreatic cancer. The Fig. 7. exhibits the dataset data. This figure shows 590 patients sample_id, patient_cohort,sample origin,age,sex,diagnosis, 199 stages, 208 diagnosis samples, 350 plasma tests, 590 cretinine, biomarkers are presented in the dataset.

## Sample code and implications

**Figure 8:**
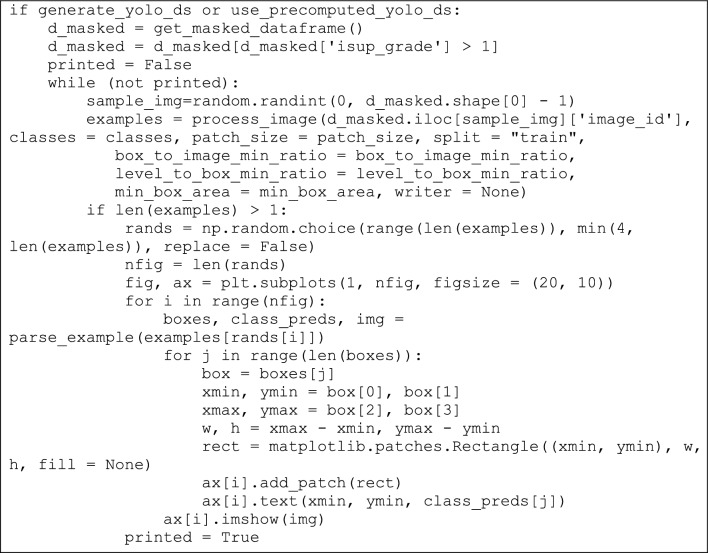
YOLOv3 code with detection strategy.

In this section sample codes are discussed. After computing threshold values, the YOLOv3 and DarknetRes is computed in python environment. Code snippets of YOLOv3 model, which is used to detect the tumour region based on computation value, is shown in Fig. 8. Finally, DarknetRes model used for classification. The few samples are shown below.

**Figure 9:**
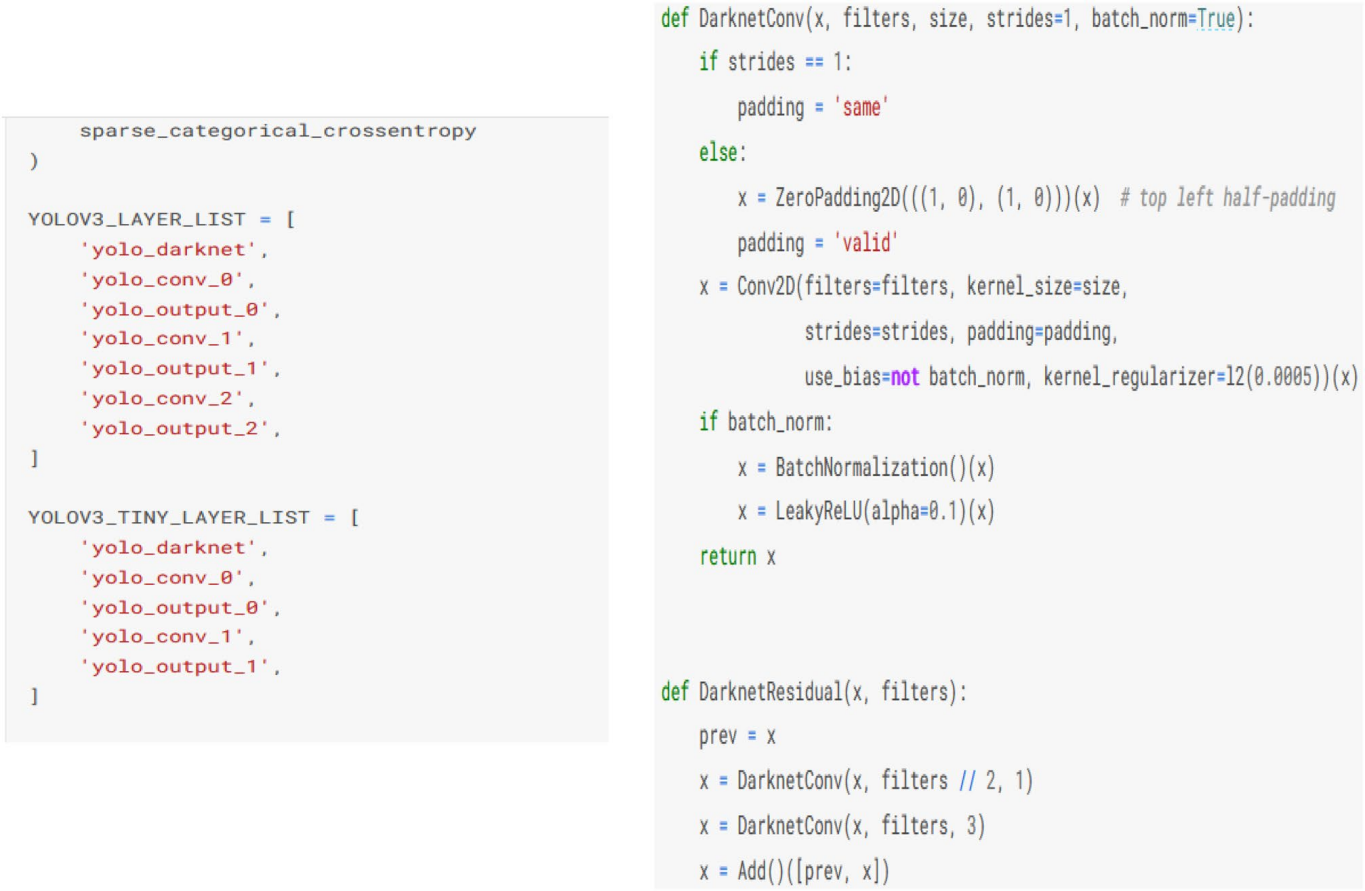
Sample snippets of YOLO layer and DraknetRes computation.

**Figure 10:**
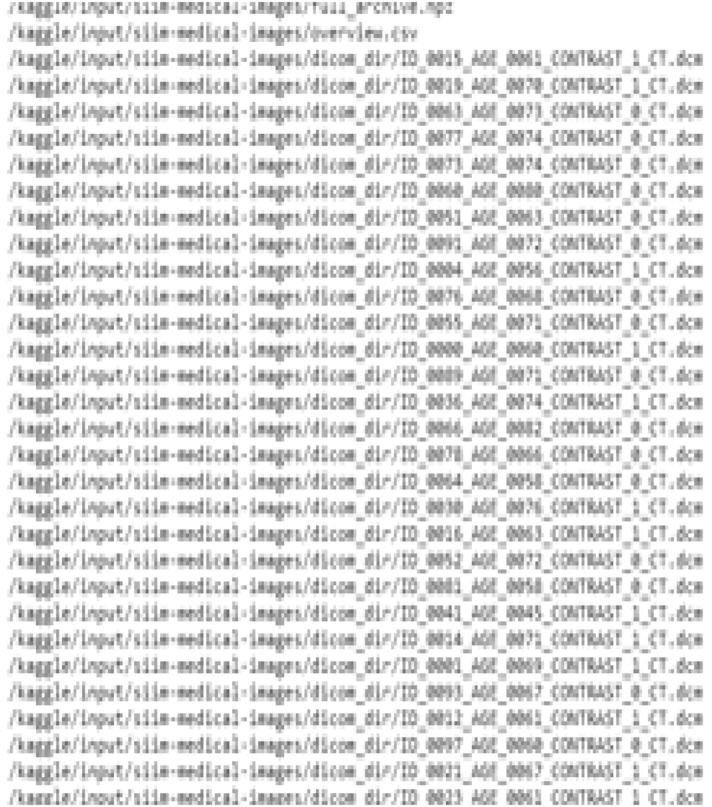
Input loading.

**Figure 11:**
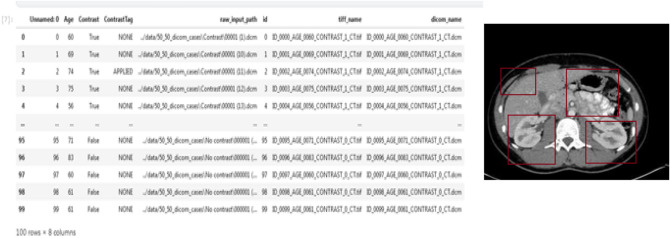
Prediction values and sample predicted output.

**Figure 12:**
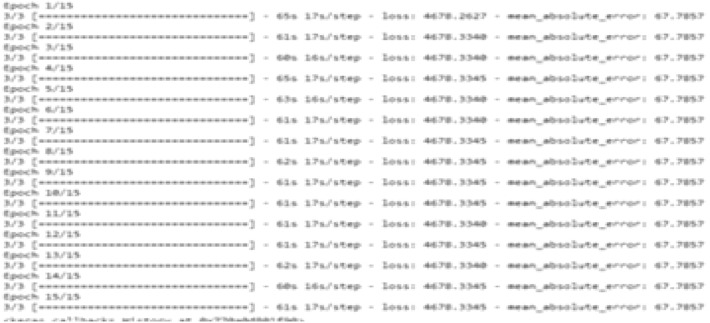
Error value computation.

Figure 9 shows sample coding snippets for the YOLO and DarknetRes neural networks. in Fig. 10 you can see how input is loaded in the platform to compute. The predicted output values are shown in the Fig. 11 and error value computation are shown in the Fig. 12. However algorithm computes for 11 min and does not fail in any instances during the computation. From confusion matrix below it can be confirmed that the proposed model predicts data with high accuracy level.

## Result and discussion

Using the two datasets, we tested our method for both binary and ternary classifications, and we determined how well it performed using the standard measures for such endeavours as accuracy, precision, and recall. The proportion of correctly supervised classification (abbreviated ${TR}_{PS}$) is used to quantify the quality of a picture. The accuracy of class K is defined as the fraction of pictures properly labelled as belonging to class K (denoted ${TR}_{PS}$) relative to the total number of images labelled as belonging to class (${TR}_{PS}$ + FPi). Among all the photos that should be identified as class K, the quantity of those that are properly classified as class K (signified as ${TR}_{PS}$) is the recall for class K. The following is how these measurements are made:

9 $$Accuracy ( AC)=\frac{{TR}_{PS}}{{TR}_{PS}+{TR}_{NG}+{FA}_{PS}+{FA}_{NG}}$$

**Figure 13:**
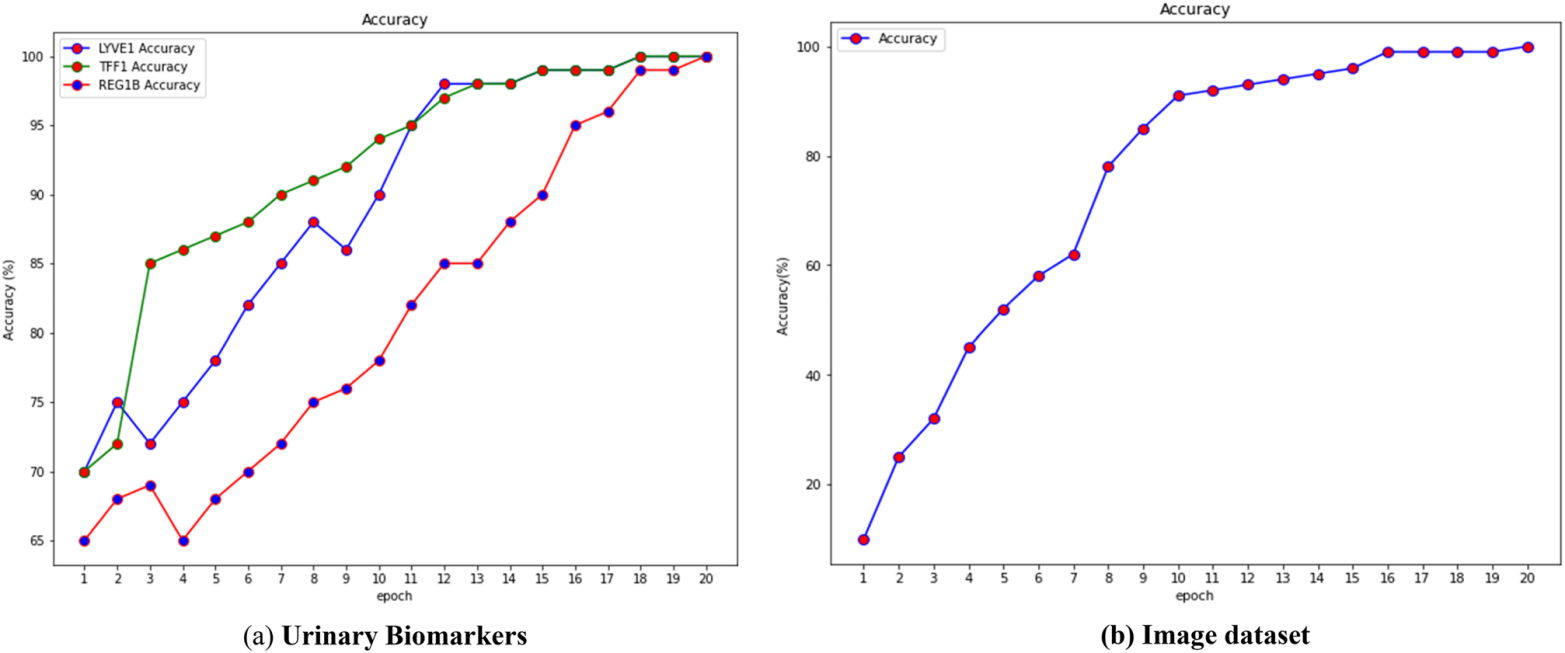
Accuracy on the datasets.

The Fig. 13 depicts the accuracy on the dataset Urinary Biomarkers and also on the image dataset. Both dataset produces the accuracy nearlyb100% on classifying the pancreatic cancer.

10 $$Precision (PR)=\frac{{TR}_{PS}}{{TR}_{PS}+{FA}_{PS}}$$

11 $$Recall(RC)=\frac{{TR}_{PS}}{{TR}_{PS}+{FA}_{NG}}$$

12 $$F1-Score(F1)=\frac{2\times Precision (PR)\times Recall(RC)}{Precision (PR)+Recall(RC)}$$

**Figure 14:**
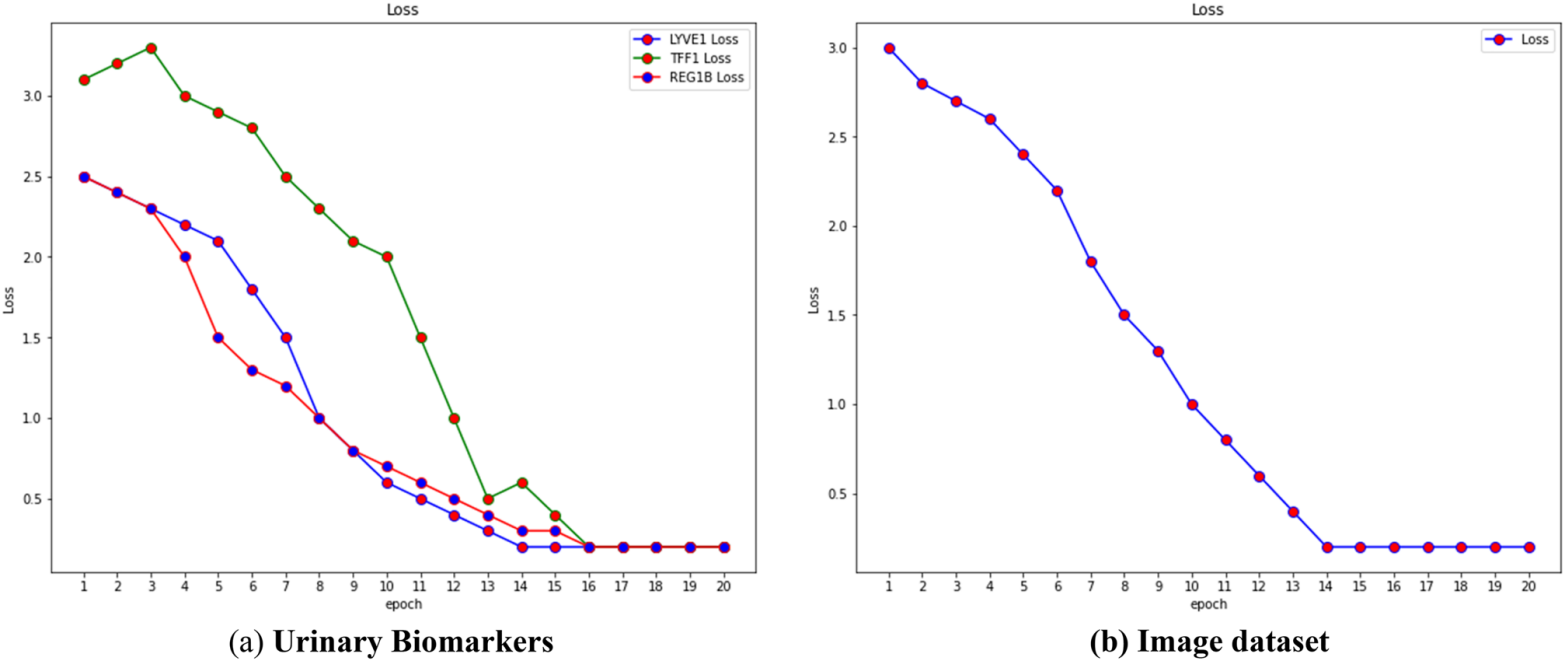
Loss on the dataset.

Given that accuracy evaluates a classifier's performance across all classes and not just for a single class Ci, it was the primary metric we used to assess the efficacy of our method. The Fig. 14. Shows the loss of the model on both datasets.

In cancer diagnosis, sensitivity equals recall, or the ratio of properly predicted malignant lesions to total malignant lesions.

13 $$Sensitivity(SE)=Recall(RC) in cancer detectection = =\frac{{TR}_{PS}}{{TR}_{PS}+{FA}_{NG}}$$

Specificity in detecting non-cancer cases is the Recall in detecting non-malignant cases which is the properly predicted non-malignant instances divided by the all-non-malignant cases);

14 $$Specificity(SP)=Recall(RC) in non-cancer detectection = =\frac{{TR}_{NG}}{{TR}_{NG}+{FA}_{PS}}$$

Accuracy in cancer diagnosis is measured by the percentage of malignant lesions for which a correct prediction was made, relative to the overall number of malignant lesions.

**Figure 15:**
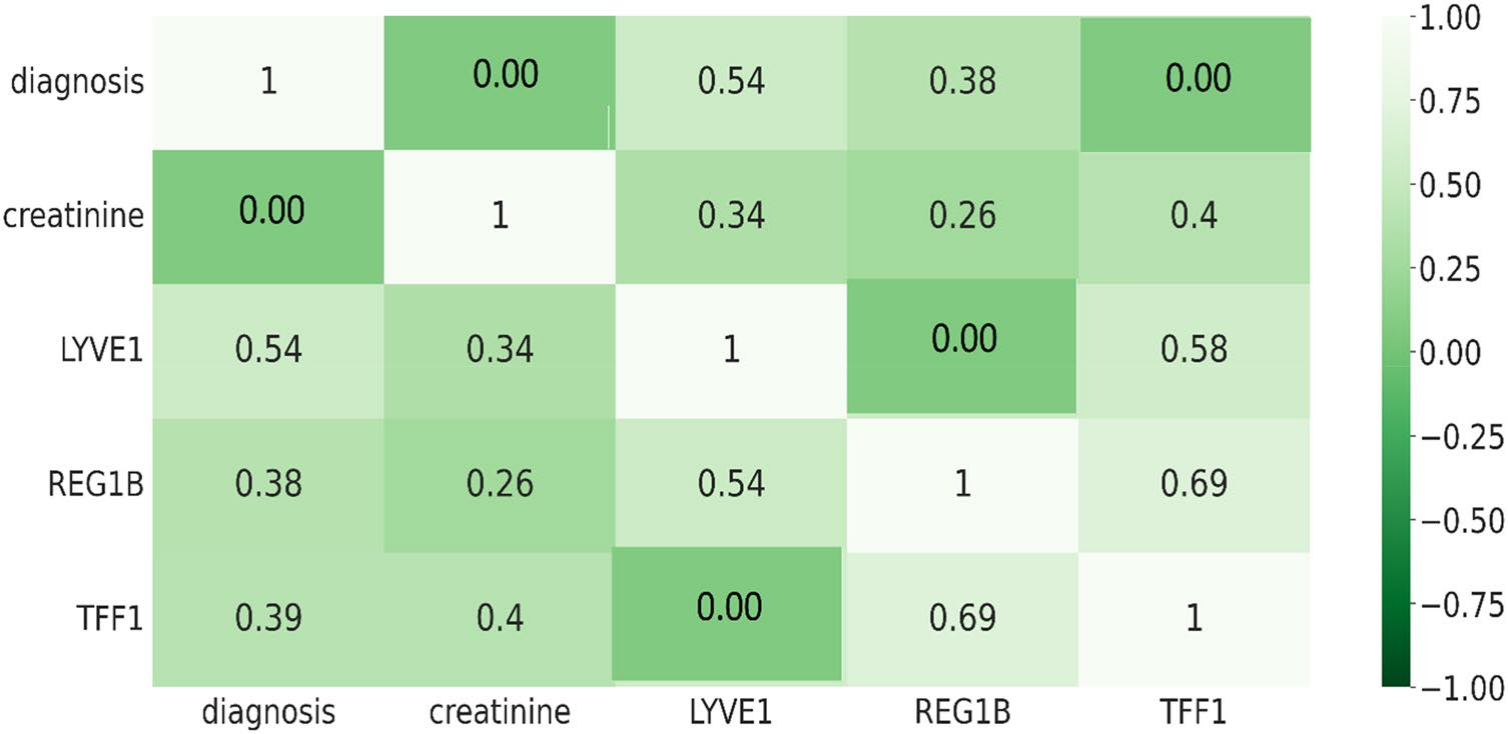
Confusion matrix on the dataset Urinary Biomarkers.

The Fig. 15 shows confusion matrix for urinary biomarker dataset. Creatinine biomarker was predicted as Creatinine with 100% accuracy. However, Creatinine biomarker has mispredicted as LYVE1 as 34%, REG1B as 26%, TFF1 as 40%. LYVE1 biomarker has mispredicted as Creatinine as 34% and TFF1 as 58%. REG1B biomarker has mispredicted Creatinine as 26%, LYVE1 as 54% and TFF1 as 69%. TFF1 biomarker has mispredicted Creatinine as 40%, REG1B as 69%.

**Figure 16:**
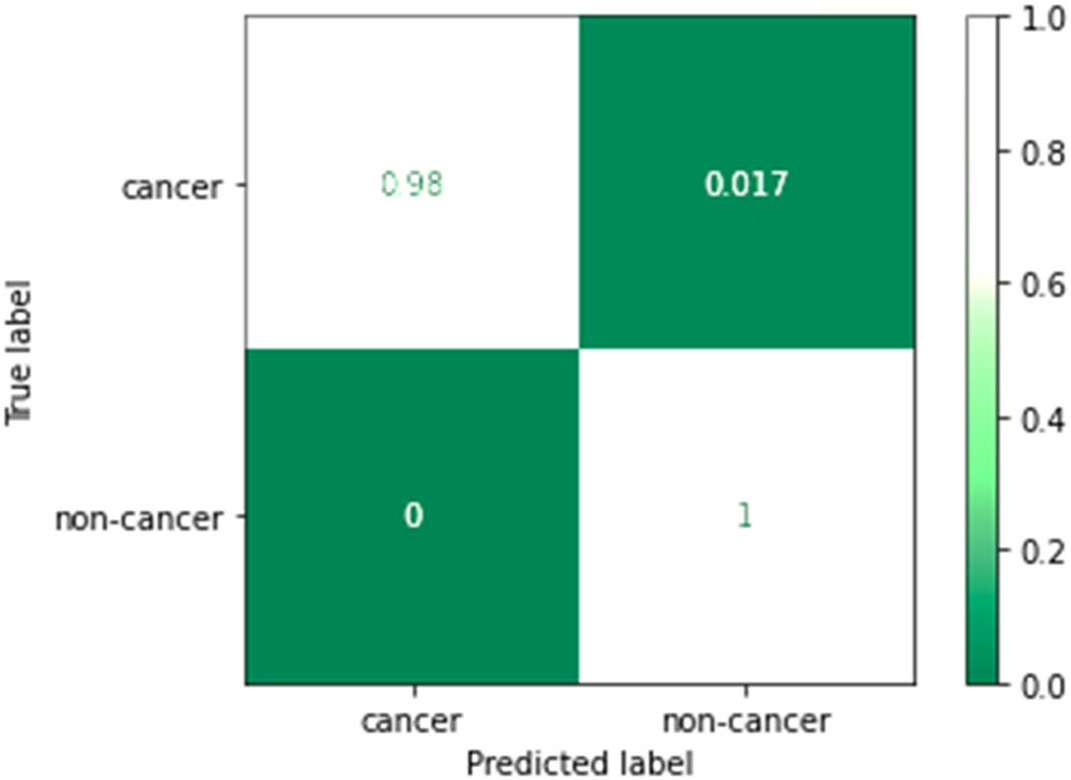
Confusion matrix on the CT image dataset.

Figure 16 shows the confusion matrix of the non-cancer and cancer result predictions for image dataset. The confusion matrix confirms that the model achieves the 100% accuracy on Urinary Biomarkers dataset and 99.9% accuracy on the CT image dataset.

The Table 2 predicts the precision recall and the f1-score produced by our proposed YCNN model. The results ensures that the proposed model gives the 100% accuracy on classification.

**Table 2:** Performance evaluation of the model YCNN.

	Precision	Recall	f1-score
1	100%	100%	100%
2	98%	100%	99%
3	100%	99%	98%
Accuracy			100%
Macro avg	100%	100%	100%
Weighted avg	100%	100%	100%

**Figure 17:**
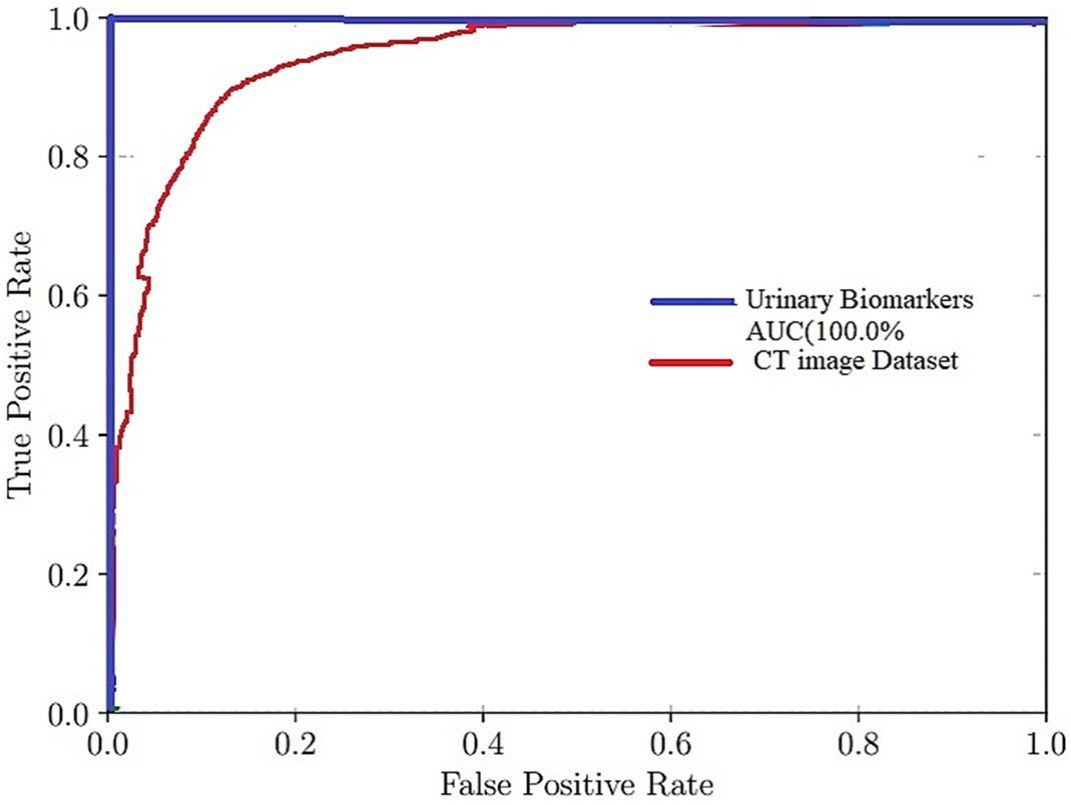
AUC curve of the proposed model.

The domains of illness diagnosis and treatment, care coordination, medication research and development, and precision medicine stand to benefit greatly from the use of machine learning. Its applicability to seeing the pancreas has been hampered since the pancreatic is very changeable in shape, size, and position, but it only occupies a very small percentage of a CT picture. This makes it difficult to observe the pancreas. As a direct consequence of this, diagnostic efficiency and accuracy have been subpar. The model consists of four stages: image screening, the localization of the pancreas, the division of the pancreas, and the diagnosis of pancreatic tumours. It achieves an area under the curve (AUC) of 1.00, an F1 score of 99.9%, and an accuracy of 100.0% on an independent testing dataset. The Fig. 17 shows the accuracy of AUC.

The creation of a more comprehensive dataset that includes a wider variety of pancreatic tumours than was previously possible as a result of this work is yet another advantage of the study. This may be of assistance to the deep learning system when it comes to recognising photos of the various pancreatic tumour kinds. The model can distinguish between the many cancers that can occur in the pancreas. The end-to-end automatic diagnosis is another strength of the system. This type of diagnosis takes only about 16.5 s per patient to complete, beginning with the input of the initial abdominal CT image and ending with a diagnosis result. It has great diagnostic and curative promise since it can manage and substantially interpret huge volumes of data fast, correctly, and affordably in clinical settings. For instance, the model might be utilised for large-scale pre-diagnosis during physical examinations, or it could be used to aid with diagnosis at low-level facilities that have limited resources. One more feature of the model that has the potential to help improve its reliability is its capacity to generate saliency maps, which can be used to pinpoint the aspects of diagnostic decision making that are of the utmost significance. Despite the fact that our method relies solely on evidence obtained from CT scans, medical professionals have access to additional information, such as the medical histories of patients and their testimonies. Consequently, the decision making of independent practitioners, and not just the results of a deep learning system, should proceed to be the basis for convincing symptoms and care planning. We conducted the same research on the other baseline model such as VGG, DenseNet, etc. The Table 3 displays the promising outcomes of the performance judgement. For instance, the MobileNet achieves nearly 99% but not more than YCNN model.

**Table 3:** Accuracy comparative analysis with other models.

Model	Urinary biomarkers accuracy (%)	Image dataset accuracy (%)
MLP	75.8	80.6
LSTM	85.9	88.5
CNN	96.8	97.5
VGG19	98.9	98.5
Resnet50	97.5	98.6
Inception	96.5	97.8
DenseNet	98.6	98.4
MobileNet	98.8	99.5
YCNN	100	100

The results presented in the Table 3 indicate the performance of different models for pancreatic cancer detection using urinary biomarkers and image datasets. Some useful insights that can be derived from presented results are as follows:

1. *Urinary Biomarkers vs. Image Dataset Accuracy* The table shows that the accuracy of urinary biomarkers is generally lower than that of image datasets. This suggests that image-based diagnostic tests might have a higher predictive power for pancreatic cancer detection compared to urinary biomarkers alone.
2. *Model Performance* The table includes various models used for pancreatic cancer detection. Some models, such as YCNN, achieve perfect accuracy (100%) for both urinary biomarkers and image datasets. These models demonstrate the potential for highly accurate detection of pancreatic cancer.
3. *Performance Variations among Models* There are notable variations in performance among different models. For instance, MLP and LSTM models achieve lower accuracy compared to other models. On the other hand, CNN-based models, including VGG19, Resnet50, Inception, and DenseNet, show higher accuracy for both urinary biomarkers and image datasets. MobileNet also performs well in terms of accuracy. These results suggest that convolutional neural network (CNN)-based models have the potential to be effective tools for pancreatic cancer detection.
4. *Potential Clinical Applications* The strong performance of certain models, especially those based on CNN architecture, indicates their potential for clinical application in pancreatic cancer detection. These models could be integrated into computer-aided diagnostic systems to assist healthcare professionals in making more accurate and timely diagnoses. However, further research and validation studies are necessary before implementing these models in clinical practice

## Conclusion and future work

In the realm of medicine, deep learning has contributed to developments in the analysis and forecast of a wide variety of disorders. In this study, a novel automated YCNN was presented to help the pathologist classify pancreatic cancer grades based on pathological images. The pycharm and Google Colab platforms serve as the backbone of the system, which also includes the YOLO model for making predictions based on the data. Input pictures are scaled up and divided into 224 × 224 pixel patches before being fed into the YCNN all at once. After that, CNN is used to categorise the patches into their respective grades, and then the patches are stitched back together to produce a single complete image before the final result is sent to the pathologist. On the datasets, f1-score measures of 0.99 and 1.00 have been reported, which are both encouraging. The proposed system could be enhanced by adopting the most advanced deep learning model, expanding the image dataset, and using augmentation to improve the learning rate of the model on a variety of colour variations. In addition, a more recent method of synthetic image generation, can be planned to generate more images of pancreatic cancer pathology with the guidance of specialists before the training process begins. At this point, the findings of this research may be able to assist in giving pathologists a consistent diagnosis for the grade of pancreatic cancer by making use of a straightforward web interface that does not require any installation. In the future, we anticipate that the system will provide the pathologist with a second opinion if it is able to improve its performance and achieve an accuracy that is closer to 1.

## Data Availability

The datasets generated during and/or analysed during the current study are available in the [kaggle] repository, [https://www.kaggle.com/datasets/kmader/siim-medical-images?select=dicom_dir], [https://www.kaggle.com/code/kerneler/starter-pancreas-ct-dataset-628d2558-a/data], [https://www.kaggle.com/datasets/johnjdavisiv/urinary-biomarkers-for-pancreatic-cancer].
